# Violence against caregivers of older adults with chronic diseases is associated with caregiver burden and depression: a cross-sectional study

**DOI:** 10.1186/s12877-022-02950-7

**Published:** 2022-03-30

**Authors:** Kanokporn Pinyopornpanish, Winita Wajatieng, Niphaporn Niruttisai, Nida Buawangpong, Nopakoon Nantsupawat, Chaisiri Angkurawaranon, Wichuda Jiraporncharoen

**Affiliations:** 1 Department of Family Medicine, Faculty of Medicine, Chiang Mai University, 110 Inthawarorot Rd., Sriphum, Muang, Chiang Mai, 50200 Thailand; 2 Global Health and Chronic Conditions Research Group, Chiang Mai University, Chiang Mai, Thailand; 3 Nakornping Hospital, Chiang Mai, Thailand; 4 Uthai Thani Hospital, Uthai Thani, Thailand

**Keywords:** Violence, Abuse, Caregiver, Older adults, Elderly

## Abstract

**Background:**

Caregivers play a vital role in caring for the aging population, however the occurrence of violence against the caregiver is an increasing area of concern. This study aimed to investigate the prevalence of violence against the primary caregivers of community dwelling older adults with chronic diseases, and to determine the factors associated with violence and its association with caregiver outcomes.

**Methods:**

A cross-sectional study was conducted. HITS questionnaire, the 22-item Zarit Burden Interview and Patient Health Questionnaire-9 were used to assess violence against caregiver, caregiver burden and depression, respectively.

**Results:**

Out of 123 caregivers of older adults, the overall prevalence of violence was 28.46%. Independent variables which could be the protective factors for violence against caregiver included higher ADL, older age of caregiver, and being a relative. The patient characteristic that is a potential risk factor for violence against caregiver was having cancer as a principal diagnosis. Statistically significant associations were found between violence and caregiver burden (aOR 4.94, p 0.004) and depression (aOR 7.03, p 0.006).

**Conclusion:**

Violence against caregivers of older adults is not uncommon. Experiencing violence was found to be associated with caregiver outcomes including depression and caregiver burden. Therefore, this important issue must not be ignored.

## Background

Interpersonal violence is defined as the intentional use of physical force or power, threatened or actual, against other persons by an individual or small group of individuals [1]. In a growing body of literature, there are increasing reports about violence against caregivers. This is especially the case among those caregivers who take care of people with chronic conditions such as mental health conditions. An association has been verified between mental health patients and violent behavior directed towards their carer [2]. It has been found that nearly 90% of the caregivers experienced psychological violence and nearly 80% experienced physical violence perpetrated by the patients with schizophrenia [3]. In a study about caregivers of patients with severe mental illness, around 70% of primary caregivers suffered from violence, 60% experienced verbal abuse and nearly 50% experienced physical abuse [4]. While report about violence against carers of people with other chronic diseases was limited, aggressive behavior among people with chronic conditions has been reported, for example, in patient with neurocognitive disorder [5, 6] and cancer [7].

In providing care to an aging population many of whom suffer from chronic diseases, a need which is increasing worldwide, the needs of the caregiver [8], including issues about violence cannot be overlooked. Evidence shows that more than half of older adults, those aged between 85 and 90 years, need family caregivers due to their limitations in physical health and functional impairment and the older the age the greater the need of assistance [9, 10]. While many studies have focused on the caregiver as a perpetrator of abuse of older adults (elder abuse) [11–14], little is known about violence against the caregiver for this population. There was a study carried out in Turkey in nursing home staffs who work with elderly residents [15]. It was found that 56% experienced violence, mostly taking the form of verbal abuse, and less than 10 % received medical or psychological support. Another study about violence towards staff in a nursing home in Sweden found that about 11% experienced violence and it can be repetitive. A variety of emotional reactions when exposed to violence were recorded, for example, aggression, astonishment, antipathy, or fear. One-third of carers had physical consequences, including wounds and bruising [16]. This supports the concept that violence could be harmful to both the physical and mental health of caregivers.

It is fully accepted that caregivers play an important role in caring for aging population, however, there needs to be an awareness concerning violence against the caregivers. This is not only true in a nursing home setting, but also in the community dwelling population, as it could affect caregiver outcome and cause caregiver burnout [17, 18] which might impact the quality of care. Patients who tend to have aggressive behavior, for example, patients with Alzheimer’s disease with severe behavioral problems, were found to be associated with caregiver burden and depression in caregivers [19]. Evidence pertinent to this issue is still lacking in developing countries such in Thailand, despite it have one of the most rapidly increasing ageing population in the developing world. Therefore, this study aimed to investigate the prevalence of violence against caregivers of community dwelling older adults with chronic diseases, and to determine the factors associated with violence and its association with caregiver outcomes.

## Methods

A cross-sectional study was conducted by interviewing the primary caregiver of each older adults with chronic disease who received home health care service from two urban primary care units in Chiang Mai province, Thailand (Nakornping Hospital and Maharaj Nakorn Chiang Mai hospital) during July 2016 – March 2017. The convenience sampling was used. A primary caregiver refers to the individual who self-identifies as having the primary responsibility for providing care and being involved in the activities in daily self-care or health care of the older adult care recipient. General information about patients and caregivers was accumulated. Additional standard questionnaires were used to determined patient functional status, social support, violence against caregiver, caregiver burden and depression.

### Barthel activities of daily living index

The Thai-version of Barthel Activities of Daily Living index was used to assess activities of daily living (ADL) of the patient. It consists of 10 basic domains including feeding, hygiene, transfer, toileting, ambulation, dressing, stairs, bathing, and continence (stool and urine) [20]. The scores range from 0 to 20. A higher score of ADLs indicates better daily function.

### Multidimensional Scale of Perceived Social Support (MSPSS)

The Thai-version of MSPSS, a 12-item questionnaire, was used to measure perceived social support from three sources: family, friends, and others [21]. The 7-response Likert scale ranges from 1 (very strongly disagree) to 7 (very strongly agree). The total scores range from 12 to 84. The higher score means a higher perception of social support.

### HITS questionnaire

This self-reporting questionnaire was adapted from the original version by Sherin et al. [22]. In this version we asked, “how often has the care recipient abused (hurting, insulting, being threaten, or screaming at) the participant within a 12-month period?” The 5-response Likert scale ranges from 1 (never) to 5 (frequently); the total score is 4-20. The higher score indicates the higher the level of abuse by their care recipient. We used the cutoff of 5 to define an experience of violence. The factor loadings for the four items ranged from 0.73 to 0.91. Reliability statistics were performed on four items, resulting in a Cronbach’s Alpha coefficient of 0.84 which indicates a high level of reliability.

### The 22-item Zarit Burden Interview (ZBI-22)

The Thai-version of ZBI-22 was used to assess caregiver burden [23]. The 5-response Likert scale ranges from 0 (never) to 4 (nearly always); the total scores range from 0 to 88. The higher scores imply the higher the feeling of burden. The cutoff score of 20/21 was used to differentiate between the non-burden and burden population.

### Patient health Questionnaire-9 (PHQ-9)

The PHQ-9 is used to measure depressive symptoms [24]. The 4-response Likert scale ranges from 0 (not at all) to 3 (nearly every day); the total scores range from 0 to 27. The higher the score the higher the level of depression. The cutoff score of 6/7 was used to differentiate between the non-depressed and depressed population.

### Statistical analysis

The descriptive data are displayed as frequencies and percentages for categorical data, mean and standard deviation (SD) for normally distributed continuous data, and median and interquartile range (IQR) for non-normally distributed continuous data. Shapiro –Wilk W test was used to test for normality of continuous variables. We used logistic regression analysis to assess the association between independent variables and experience of violence. Factors which showed potential association from the univariable analysis were recruited into the final model (*p*-value < 0.2). To assess the association between experience of violence and caregiver outcomes (caregiver burden and depression), we used multivariable logistic regression analysis by including caregiver age, sex, income, disease, relationship, time of care, patient ADL, and social support as covariates. A *p*-value of less than 0.05 was considered to have statistical significance. All analyses were performed using STATA SE 15.1.

## Results

More than half of the older adults were female (55.3%) and the average age was 75.7 years (SD 9.8). The majority of participants had neurological diseases (stroke and dementia) as a primary disease (63.4%). Out of a total of 123 caregivers of older adults with chronic diseases, the majority of the caregivers were female (74.0%) and the average age was 57.3 (SD 15.0). The demographic data of patients and caregivers are shown in Table 1.

**Table 1:** Characteristics of patients and caregivers

	Total *N* = 123
**Patient characteristics**	
Female, n (%)	68 (55.3)
Age (years), mean ± SD	75.7 ± 9.8
Category of principal diagnosis, n (%)	
- Neurological diseases	78 (63.4)
- Heart diseases	15 (12.2)
- Cancer	11 (8.9)
- Musculoskeletal diseases	12 (9.8)
- Kidney diseases	5 (4.1)
- Respiratory disease	1 (0.8)
- Endocrine disease	1 (0.8)
ADL, mean ± SD	11.3 ± 7.5
**Caregiver characteristics**	
Female, n (%)	91 (74.0)
Age (years), mean ± SD	57.3 ± 15.0
Having a partner, n (%)	86 (69.9)
Having underlying disease(s), n (%)	78 (63.4)
Education secondary school and higher, n (%)	77 (62.6)
Employed, n (%)	81 (65.9)
High personal income (> 15,000 THB), n (%)	41 (33.6)
Relationship, n (%)	
- Hired caregiver	13 (10.6)
- Child	41 (33.3)
- Spouse	52 (42.3)
- Sibling	7 (5.7)
- Others	10 (8.1)
Time carrying out care per day (hours), median (IQR)	12 (5 – 24)
Duration of care (months), median (IQR)	60 (24 - 120)
HITS score	
- median (IQR)	4 (4 – 5)
- Total score ≥ 5, n (%)	35 (28.5)
ZBI-22, median (IQR)	16 (7 - 35)
MSPSS, median (IQR)	61 (48 – 72)
PHQ-9, median (IQR)	2 (1 - 6)

*Abbreviations*: *ADL* activities of daily living, *IADL* instrumental activities of daily living, *IQR* interquartile range, *SD* standard deviation, *ZBI-22* The 22-item Zarit burden interview, *HITS* Hurt-Insult-Threat-Scream, *MSPSS* Multidimensional Scale of Perceived Social Support, *PHQ-9* Patient Health Questionnaire-9

From Table 1, the overall prevalence was 28.5. Table 2 shows the self-reported details of experiencing of violence by care recipients. All types of violence were reported by caregivers; however, the frequency was not high. While they were less likely to report physical assault (5.7%), screaming was the most common factor reported by caregivers (22.7%).

**Table 2:** Details of violence against caregiver (HITS questionnaire)

	1 (never)	2 (rarely)	3 (sometimes)	4 (fairly often)	5 (frequently)
Hurt	116 (94.3)	5 (4.1)	2 (1.6)	0	0
Insult	106 (86.2)	9 (7.3)	6 (4.9)	1 (0.8)	1 (0.8)
Threat	114 (92.7)	4 (3.3)	3 (2.4)	1 (0.8)	1 (0.8)
Scream	95 (77.3)	17 (13.8)	9 (7.3)	1 (0.8)	1 (0.8)

Table 3 shows that independent variables which were the potential protective factors for violence against caregiver included higher ADL, older age of caregiver, and being a relative. The patient characteristic that is a potential risk factor for violence against caregiver was having cancer as a principal diagnosis. Table 4 shows the association between violence and caregiver burden (aOR 4.94, *p* 0.004) and depression (aOR 7.03, *p* 0.006).

**Table 3:** Factors associated with violence against caregivers

	Univariable analysis		Multivariable analysis	
	cOR	*p*-value	aOR	*p*-value
**Patient characteristics**				
Female	0.58	0.180	0.39	0.065
Older age	0.97	0.157	0.98	0.429
Higher ADL	0.96	0.106	0.93	0.016
Principal diagnosis: Neurological diseases	1.15	0.739		
Principal diagnosis: Heart diseases	0.59	0.443		
Principal diagnosis: Cancer	3.43	0.055	7.08	0.011
Principal diagnosis: Musculoskeletal diseases	0.47	0.350		
**Caregiver characteristics**				
Female	0.68	0.390		
Older age	0.96	0.005	0.96	0.021
Education secondary school and higher	0.86	0.707		
Employed	0.99	0.984		
High personal income (> 15,000 THB)	0.49	0.115	0.48	0.177
Being a relative	0.47	0.069	0.34	0.036
Time carrying out care per day (hours)	1.01	0.823		
Duration of care (months)	1.00	0.474

**Table 4:** Association between experience of violence and caregiver burden and depression

	Univariable analysis		Multivariable analysis^a^	
	cOR	*p*-value	aOR	*p*-value
**Burden**	4.83	< 0.001	4.94	0.004
**Depression**	3.65	0.004	7.03	0.006

^a^adjusted for caregiver age, sex, income, disease, relationship, time of care, patient activity of daily living, and social support

## Discussion

This study found that violence against caregivers was also common among those taking care of older care recipients. Even though the prevalence is around one third of the study sample, experiencing violence was found to be associated with caregiver outcomes including depression and caregiver burden. Therefore, this issue must not be overlooked.

The prevalence of violence against the caregiver in our study was 28.5%. When compared to prior study which conducted among nursing home staff caring for elderly, the prevalence in our study was lower [15, 16]. The prevalence of violence against family members who were taking care of relatives with schizophrenia in Japan were a lot higher when compared to our study [3]. In that study the incidence of psychological violence was 87.7% and physical violence was 75.8%. The prevalence of violence against caregivers of people with severe mental illness in China was also higher (74.0%) [4], 61.5% of this group experienced verbal attack and 45.2% experienced physical attack. These differences in prevalence could be associated with the care setting, the disease of the care recipient, and the different assessment tools. Or it could be the characteristic gentleness of the Thai population. Therefore, they are not easily complaint leading to the low prevalence in our study. Physical abuse in our study was 5.7% which was low but not absent. This reminds physician to be aware of harmful situations that might occur in the family.

The patient factor related to the higher levels of abuse against caregiver was having cancer as a principal diagnosis. The literature shows that violent behavior in cancer patient is not an uncommon phenomenon but is rarely addressed [7]. The direct effect from cognitive limitations, which could be from the disease itself or side effect of medication, was mentioned. Tumors located in specific brain regions including the frontal lobe, limbic system, and cerebellum are also found to be associated with aggressive personality [25]. Furthermore, indirect effect from emotional instability and anxiety during the life-change experience could cause aggression [26]. Therefore, searching for the cause of personality change and providing support for patients to cope with their emotional stress might be useful to reduce violent behavior of cancer patient.

Factors associated with lower levels of abuse included lower ADL, younger age of the caregiver, and not being a relation. Lower ADL can be related to cognitive impairment, as in dementia, which is sometimes related to unpleasant neuropsychiatric behaviors, including agitation and aggression [27]. It was reported that 59.8% of nursing home staff were abused during an ADL situation [16]. Behavior is frequently associated with the ability to communicate in older adults with impaired cognition who may have lost other means of expressing needs or unmeet expectations [28]. Younger age caregivers may not be a close relative to the older care recipient or be of a different generation. Not being related often means that the carer is employed, or a social-services caregiver and they may not be familiar with what the patient needs or does not understand the patient well. Also, there would be no emotional bond which is present if the carer is a relative.

Not surprisingly, being abused can be related to increasing depression and caregiver burden. This has also been mentioned in prior studies among caregivers of people with dementia [28] and mental health illness [4]. Carers who are exposed to violence would experience many turbulent emotions such as anger, shame, guilt, self-blame and perception of professional incompetence [16]. This would affect the relationship between the caregiver and care recipient and lead to poorer care quality. To prevent this, when involved in the care of older adults, physicians should look for, not only violence against the older adults, but also violence against the caregiver. Once the event has been detected, the situation requires immediate management. Apart from the provision of treatment for any emergency condition (consequences of physical abuse), the caregiver should be asked to explore the reasons why they were abused, and given training in how to handle abusive older adults.

Evidence suggests that the reasons for violent behavior are complex and multifactorial [29]. These factors include patient characteristics (diseases and somatic symptoms), inappropriate caregiver approach and an overstimulating environment. Caregivers should be given support in how to address these situations, making adjustments if possible. In addition, they should be made aware of how to handle an acute situation. If they are physically abused, they should know how to escape from the situation or how to call for help. If it is verbal or emotional abuse, they should be made aware of how to cope with it. For example, mindfulness skills have been used to help prevent burnout in caregivers of aggressive adults [30]. Emotion based coping strategies have been found to mitigate the perception of caregiver burden [31]. In many cases, the manner of caring for the patient could also reduce abusive behavior. If violence is a meaningful communication from a sensation of discomfort or a feeling of insecurity by the patient, a person-centered approach should be employed. Intervention should be provided individually and family by family. In caring for individuals with dementia, person-centered care planning could reduce conflict between the carer and care recipient [32].

Our study highlights the importance of the detection of violence against caregivers of older adults. However, this study is not without its limitations. First, as is frequently the limitation of a cross-sectional study, temporal relationships could not be shown. Secondly, the questionnaire does not include some types of violence such as sexual or financial abuse.

## Conclusion

Violence against caregivers is not uncommon. Our study indicates a relationship between caregiver burden and depression. Thus, it is important for physicians to look for indications of problems in the relationship between the carer and the caregiver and know how to give advice and manage the situation effectively. This is especially the case in carers at high risk including those providing care for patients with low ADL, caregivers with a younger age, and employed caregivers who may be unaware of the needs of the patient.

## Data Availability

The datasets used and/or analyzed during the current study are available from the corresponding author on reasonable request.

## Abbreviations

ADL: Activities of daily living
MSPSS: Multidimensional Scale of Perceived Social Support
PHQ-9: Patient Health Questionnaire-9
ZBI-22: The 22-item Zarit Burden Interview
